# Supplementary material for: SERPINE1 mRNA Binding Protein 1 Is Associated with Ischemic Stroke Risk: A Comprehensive Molecular–Genetic and Bioinformatics Analysis of SERBP1 SNPs
Source: Int J Mol Sci. 2023 May 13;24(10):8716. doi: 10.3390/ijms24108716 (PMC10217814; doi:10.3390/ijms24108716)
Supplement: Supplementary file 1 [file ijms-24-08716-s001.zip › ijms-2358303-supplementary.pdf]

Table S1: Distribution of *SERBP1* genotypes in ischemic stroke patients/healthy controls and their correspondence to the Hardy–Weinberg equilibrium.

SNP	Genotypes	Controls	H_o (H_e)[1]	P[2]	IS patients	H_o (H_e)[3]	P[4]
rs4655707	C/C	441 (40.7%)	0.4446 (0.4666)	>0.05	296 (37%)	0.4869 (0.4741)	>0.05
	C/T	482 (44.5%)			389 (48.7%)		
	T/T	161 (14.8%)			114 (14.3%)		
rs1058074	T/T	616 (56.8%)	0.3751 (0.3696)	>0.05	434 (52.3%)	0.3976 (0.4017)	>0.05
	T/C	407 (37.5%)			330 (39.8%)		
	C/C	62 (5.7%)			66 (8%)		
rs12561767	A/A	441 (41.6%)	0.4406 (0.4628)	>0.05	302 (37.9%)	0.4705 (0.4739)	>0.05
	A/G	467 (44.1%)			375 (47%)		
	G/G	152 (14.3%)			120 (15.1%)		
rs12566098	G/G	742 (62.3%)	0.3233 (0.3380)	>0.05	467 (53.7%)	0.3947 (0.3898)	>0.05
	G/C	385 (32.3%)			343 (39.5%)		
	C/C	64 (5.4%)			59 (6.8%)		
rs6702742	G/G	455 (41%)	0.4405 (0.4661)	>0.05	306 (38.8%)	0.4619 (0.4715)	>0.05
	A/G	489 (44%)			364 (46.2%)		
	A/A	166 (14.9%)			118 (15%)		
[1] – observed (Ho) and expected (He) heterozygosity in healthy controls; [2] – P-HWE in healthy controls; [3] – observed (Ho) and expected (He) heterozygosity in IS patients; [4] – P-HWE in IS patients							

Table S2: Results of the analysis of associations between *SERBP1* gene SNPs and ischemic stroke risk depending on sex and smoking status.

Genetic variant	Effect allele	Other allele	N	OR (95% CI)[1]	P[2]	N	OR (95% CI)[1]	P[2]
			Males			Females		
			Nonsmokers					
rs4655707	T	C	481	1.22 [0.94; 1.58]	0.14	800	1.07 [0.86; 1.33]	0.56
rs1058074	C	T	499	1.35 [1.00; 1.82]	0.054	818	**1.34 [1.06; 1.69]**	**0.02**
rs12561767*	G	A	487	**1.36 [1.05; 1.77]**	**0.02**	790	1.08 [0.87; 1.35]	0.5
rs12566098*	C	G	526	**1.49 [1.10; 2.01]**	**0.009**	874	**1.43 [1.14; 1.80]**	**0.003**
rs6702742	A	G	489	**1.33 [1.03; 1.73]**	**0.03**	816	0.96 [0.77; 1.20]	0.74
			Smokers					
rs4655707	T	C	448	0.88 [0.66; 1.16]	0.36	154	1.37 [0.70; 2.68]	0.36
rs1058074	C	T	451	0.84 [0.63; 1.13]	0.26	137	1.04 [0.49; 2.20]	0.92
rs12561767*	G	A	431	0.86 [0.65; 1.14]	0.3	149	1.49 [0.76; 2.93]	0.24
rs12566098*	C	G	500	0.98 [0.74; 1.31]	0.92	160	1.16 [0.58; 2.33]	0.67
rs6702742	A	G	450	0.86 [0.66; 1.13]	0.28	143	1.33 [0.69; 2.54]	0.39
[1] – odds ratio and 95% confidence interval; [2] – *p*-value. All calculations were performed relative to the minor alleles (effect allele). Statistically significant differences are marked in bold; tag SNPs are marked with an asterisk.								

Table S3: Established statistically significant associations of *SERBP1* genotypes with clinical and biological characteristics of IS patients.

SNP	Groups	Genotypes	N	Me [Q1-Q3]	Kruskal-Wallis-Test (P)	Mann–Whitney U test: P(FDR)
Activated Partial Thromboplastin Time (APTT)						
rs4655707	Entire group	C/C^{1}	197	34.0 [29.9-37.0]	**0.04**	P^{1-2}=0.75 (0.75)
		C/T^{2}	238	32.65 [28.3-37.0]		P^{1-3}=**0.01 (0.01)**
		T/T^{3}	80	31.0 [28.0-35.0]		P^{2-3}=**0.03 (0.045)**
rs1058074	Low fruit/ vegetable intake	T/T^{1}	155	34.0 [30.0-37.0]	**0.04**	P^{1-2}=0.26 (0.26)
		T/C^{2}	110	32.45 [29.0-37.0]		P^{1-3}=**0.01 (0.03)**
		C/C^{3}	27	30.9 [27.2-34.0]		P^{2-3}=0.10 (0.15)
rs12561767*	Entire group	A/A^{1}	203	34.0 [29.3-37.0]	**0.02**	P^{1-2}=0.75 (0.75)
		A/G^{2}	244	32.7 [29.0-37.0]		P^{1-3}=**0.007 (0.021)**
		G/G^{3}	82	31.0 [28.0-35.0]		P^{2-3}=**0.016 (0.024)**
	Low fruit/ vegetable intake	A/A^{1}	112	34.2 [29.0-37.7]	**0.01**	P^{1-2}=0.51 (0.51)
		A/G^{2}	121	32.7 [29.0-37.0]		P^{1-3}=**0.005 (0.015)**
		G/G^{3}	47	30.9 [28.0-34.0]		P^{2-3}=**0.01 (0.015)**
rs12566098*	Low fruit/ vegetable intake	G/G^{1}	164	34.0 [29.55-37.0]	**0.02**	P^{1-2}=0.29 (0.29)
		G/C^{2}	114	32.45 [29.0-37.0]		P^{1-3}=**0.005 (0.015)**
		C/C^{3}	26	30.75 [29.0-31.9]		P^{2-3}=**0.057 (0.086)**
rs6702742	Entire group	G/G^{1}	207	34.0 [30.0-37.0]	**0.04**	P^{1-2}=0.63 (0.63)
		G/A^{2}	245	33.1 [29.0-37.0]		P^{1-3}=**0.01 (0.03)**
		A/A^{3}	84	31.1 [28.5-35.0]		P^{2-3}=**0.036 (0.054)**
	Low fruit/ vegetable intake	G/G^{1}	115	34.5 [30.5-37.0]	**0.036**	P^{1-2}=0.50 (0.50)
		G/A^{2}	119	32.7 [29.0-37.0]		P^{1-3}=**0.01 (0.03)**
		A/A^{3}	49	31.0 [29.0-34.2]		P^{2-3}=**0.039 (0.059)**
BMI						
rs4655707	Normal fruit/vegetable intake	C/C^{1}	85	23.0 [22.0-24.1]	**0.038**	P^{1-2}=0.83 (0.83)
		C/T^{2}	119	23.0 [22.0-25.7]		P^{1-3}=**0.009 (0.027)**
		T/T^{3}	29	24.0 [23.0-26.7]		P^{2-3}=**0.025 (0.038)**
	BMI<25	C/C^{1}	144	22.0 [21.25-23.0]	**0.01**	P^{1-2}=0.24 (0.24)
		C/T^{2}	162	22.0 [21.0-23.0]		P^{1-3}=**0.03 (0.045)**
		T/T^{3}	53	23.0 [22.0-24.0]		P^{2-3}=**0.003 (0.009)**
rs12561767*	BMI<25	A/A^{1}	150	22.0 [21.3-23.0]	**0.003**	P^{1-2}=0.08 (0.08)
		A/G^{2}	165	22.0 [21.0-23.0]		P^{1-3}=**0.04 (0.06)**
		G/G^{3}	55	23.0 [22.0-24.0]		P^{2-3}=**0.0006 (0.002)**
rs6702742	BMI<25	G/G^{1}	151	22.0 [21.5-23.0]	**0.006**	P^{1-2}=0.08 (0.08)
		G/A^{2}	162	22.0 [21.0-23.0]		P^{1-3}=0.07 (0.07)
		A/A^{3}	55	23.0 [22.0-24.0]		P^{2-3}=**0.002 (0.002)**

Table S4: Analysis of the effect of rs4655707 *SERBP1* on the binding of DNA to transcription factors.

№	Ref/SNP аллель[1]	ТФ[2]	GAIN /LOSS[3]	Motif[4]	*p*-Value SNP impact[5]	*p*-Value Ref[6]	*p*-Value SNP[7]
1	T/C	TATA	gain	TATA_disc10	0	0.369	0.00001
2	T/C	ETS	gain	ETS_disc7	0.0001	0.063	0.0004
3	T/C	RAD21	gain	RAD21_disc5	0.0002	0.116	0.006
4	T/C	ETS	gain	ETS_disc9	0.0003	0.185	0.001
5	T/C	REST	gain	REST_disc8	0.0004	0.059	0.003
6	T/C	SP1	gain	SP1_1	0.001	0.220	0.005
7	T/C	ELF1	gain	ELF1_disc3	0.001	0.060	0.003
8	T/C	SRF	gain	SRF_disc2	0.001	0.090	0.005
9	T/C	EGR4	gain	EGR4_1	0.001	0.245	0.012
10	T/C	SP1	gain	SP1_3	0.001	0.432	0.009
11	T/C	EBF1	gain	EBF1_disc2	0.001	0.304	0.033
12	T/C	CTCF	gain	CTCF_disc8	0.001	0.224	0.028
13	T/C	EGR1	gain	EGR1_1	0.002	0.293	0.027
14	T/C	EGR2	gain	EGR2_3	0.002	0.154	0.007
15	T/C	EGR2	gain	EGR2_2	0.002	0.711	0.017
16	T/C	ESRRA	gain	ESRRA_disc2	0.002	0.802	0.017
17	T/C	ETV4	gain	ETV4_1	0.002	0.455	0.048
18	T/C	REST	gain	REST_disc9	0.003	0.122	0.017
19	T/C	MAZ	gain	MAZ_1	0.003	0.368	0.045
20	T/C	SP1	gain	SP1_4	0.003	0.149	0.008
21	T/C	ZNF143	gain	ZNF143_disc4	0.004	0.217	0.027
22	T/C	E2F1	gain	E2F1_17	0.004	0.112	0.008
23	T/C	SP1	gain	MA0079.3	0.005	0.350	0.037
24	T/C	SP1	gain	SP1_5	0.006	0.475	0.015
25	T/C	GATA	gain	GATA_disc5	0.007	0.144	0.016
26	T/C	Nr5a2	gain	MA0505.1	0.007	0.293	0.025
27	T/C	EGR3	gain	EGR3_3	0.007	0.784	0.046
28	T/C	CACBP	gain	CACBP_1	0.007	0.225	0.027
29	T/C	EGR3	gain	EGR3_2	0.007	0.060	0.006
30	T/C	ESRRA	gain	ESRRA_6	0.009	0.181	0.017
31	T/C	DMBX1	gain	TATA_disc10	0	0.369	0.00001
32	T/C	TFAP4	gain	ETS_disc7	0.0001	0.063	0.0004
33	T/C	TFAP4	gain	RAD21_disc5	0.0002	0.116	0.006
34	T/C	NHLH1	gain	ETS_disc9	0.0003	0.185	0.001
35	T/C	FIGLA	gain	REST_disc8	0.0004	0.059	0.003
36	T/C	E2F1	gain	SP1_1	0.001	0.220	0.005
37	T/C	CEBPB	gain	ELF1_disc3	0.001	0.060	0.003
38	T/C	E2F1	gain	SRF_disc2	0.001	0.090	0.005
39	T/C	Myog	gain	EGR4_1	0.001	0.245	0.012
40	T/C	GSC2	gain	SP1_3	0.001	0.432	0.009
41	T/C	EGR1	gain	EBF1_disc2	0.001	0.304	0.033
42	T/C	NFKB1	gain	CTCF_disc8	0.001	0.224	0.028
43	T/C	OTX2	gain	EGR1_1	0.002	0.293	0.027

44	T/C	TFCP2	gain	EGR2_3	0.002	0.154	0.007
45	T/C	RXRA	gain	EGR2_2	0.002	0.711	0.017
46	T/C	ESRRB	gain	ESRRA_disc2	0.002	0.802	0.017
47	T/C	EGR4	gain	ETV4_1	0.002	0.455	0.048
48	T/C	RXRA	gain	REST_disc9	0.003	0.122	0.017
49	T/C	OTX1	gain	MAZ_1	0.003	0.368	0.045
50	T/C	TCF3	gain	SP1_4	0.003	0.149	0.008
51	T/C	NR2F6	gain	ZNF143_disc4	0.004	0.217	0.027
52	T/C	ZEB1	gain	E2F1_17	0.004	0.112	0.008
53	T/C	GTF2I	gain	MA0079.3	0.005	0.350	0.037
54	T/C	NFE2	gain	SP1_5	0.006	0.475	0.015
55	T/C	SP1	gain	GATA_disc5	0.007	0.144	0.016
56	T/C	REST	gain	MA0505.1	0.007	0.293	0.025
57	T/C	SMAD3	gain	EGR3_3	0.007	0.784	0.046
58	T/C	E2F	gain	CACBP_1	0.007	0.225	0.027
59	T/C	TFAP4	gain	EGR3_2	0.007	0.060	0.006
60	T/C	SREBF1	gain	ESRRA_6	0.009	0.181	0.017
61	T/C	HNF4	loss	HNF4_4	0.002	0.019	0.216
62	T/C	PAX5	loss	PAX5_disc4	0.002	0.020	0.374
63	T/C	CREB1	loss	CREB1_2	0.004	0.023	0.520
64	T/C	TFAP4	loss	TFAP4_2	0.004	0.009	0.126
65	T/C	Tcf12	loss	MA0521.1	0.004	0.020	0.202
66	T/C	RXRB	loss	RXRB_2	0.005	0.040	0.278
67	T/C	Gfi1b	loss	MA0483.1	0.005	0.024	0.328
68	T/C	AP1	loss	AP1_disc6	0.005	0.018	0.465
69	T/C	ZBTB7A	loss	ZBTB7A_disc2	0.006	0.013	0.205
70	T/C	DPRX	loss	DPRX_2	0.006	0.039	0.988
71	T/C	OTX1	loss	OTX1_3	0.006	0.022	0.881
72	T/C	PTF1A	loss	PTF1A_1	0.006	0.044	0.249
73	T/C	BCL	loss	BCL_disc7	0.007	0.008	0.123
74	T/C	GFI1	loss	GFI1_2	0.007	0.029	0.595
75	T/C	BHLHE23	loss	BHLHE23_1	0.008	0.044	0.915
76	T/C	NR2F1	loss	NR2F1_2	0.009	0.014	0.134
77	T/C	ASCL2	loss	ASCL2_1	0.009	0.039	0.339
78	T/C	TCF12	loss	TCF12_disc3	0.009	0.005	0.062
79	T/C	FOXK1	loss	FOXK1_2	0.009	0.049	0.431
80	T/C	NHLH1	loss	NHLH1_1	0.010	0.044	0.539
81	T/C	GATA	loss	HNF4_4	0.002	0.019	0.216
82	T/C	EGR1	loss	PAX5_disc4	0.002	0.020	0.374
83	T/C	REST	loss	CREB1_2	0.004	0.023	0.520
84	T/C	CACD	loss	TFAP4_2	0.004	0.009	0.126
85	T/C	RXRA	loss	MA0521.1	0.004	0.020	0.202
86	T/C	USF	loss	RXRB_2	0.005	0.040	0.278
87	T/C	MZF1	loss	MA0483.1	0.005	0.024	0.328
88	T/C	NR2C2	loss	AP1_disc6	0.005	0.018	0.465
89	T/C	NR5A1	loss	ZBTB7A_disc2	0.006	0.013	0.205

90	T/C	NHLH1	loss	DPRX_2	0.006	0.039	0.988
91	T/C	ZEB1	loss	OTX1_3	0.006	0.022	0.881
92	T/C	ESRRB	loss	PTF1A_1	0.006	0.044	0.249
93	T/C	PAX5	loss	BCL_disc7	0.007	0.008	0.123
94	T/C	NR2E3	loss	GFI1_2	0.007	0.029	0.595
95	T/C	MXI1	loss	BHLHE23_1	0.008	0.044	0.915
96	T/C	SP3	loss	NR2F1_2	0.009	0.014	0.134
97	T/C	SP2	loss	ASCL2_1	0.009	0.039	0.339
98	T/C	SMAD2::SMAD3::SMAD4	loss	TCF12_disc3	0.009	0.005	0.062
99	T/C	HNF4A	loss	FOXK1_2	0.009	0.049	0.431
	C[8]	Positive regulation of cysteine-type endopeptidase activity involved in apoptotic signaling pathway (GO:0006919; FDR=**0.03**);<br>Response to hypoxia (GO:0001666; FDR=**0.03**);<br>Cellular response to growth factor stimulus (GO:0071363; FDR=**0.04**).					
	T[9]	-					

1 – Reference (Ref) / alternative (SNP) allele;
2 – TF - transcription factor;
3 – Binding of TF to the reference (LOSS) / alternative (GAIN) allele;
4 – Binding sites with high affinity for TF;
5 – *p*-value statistically confirming the potential gain or loss of function of the genomic region with SNP in terms of transcription factor binding;
6 – *p*-value for assessing the binding of TF to the Ref allele;
7 – *p*-value for assessing the binding of TF to the SNP allele;
8 – Biological processes pathogenetically significant for IS, in which TFs that bind to the alternative allele are jointly involved (data from the Gene Ontology resource; http://geneontology.org/);
9 – Biological processes pathogenetically significant for AI, in which TFs that bind to the reference allele are jointly involved (data from the Gene Ontology resource; http://geneontology.org/).

Table S5: Analysis of the effect of rs1058074 *SERBP1* on the binding of DNA to transcription factors.

№	Ref/ SNP allele[1]	TF[2]	GAIN /LOSS[3]	Motif[4]	*p*-Value SNP impact[5]	*p*-Value Ref[6]	*p*-Value SNP[7]
1	C/T	PROP1	gain	PROP1_2	0.0001	0.769	0.002
2	C/T	Pdx1	gain	MA0132.1	0.0002	0.143	0.007
3	C/T	VSX2	gain	VSX2_1	0.0004	0.175	0.006
4	C/T	NOBOX	gain	NOBOX_1	0.001	0.694	0.008
5	C/T	MEIS1::HOXA9	gain	MEIS1::HOXA9_2	0.001	0.089	0.006
6	C/T	SOX10	gain	SOX10_1	0.002	0.323	0.010
7	C/T	BARHL2	gain	BARHL2_6	0.003	0.375	0.004

8	C/T	DLX5	gain	DLX5_1	0.003	0.330	0.024
9	C/T	PDX1	gain	PDX1_3	0.003	0.200	0.004
10	C/T	NKX2-1	gain	NKX2-1_3	0.003	0.552	0.021
11	C/T	TGIF1	gain	TGIF1_1	0.003	0.054	0.002
12	C/T	NKX2-6	gain	NKX2-6_1	0.003	0.293	0.011
13	C/T	ISX	gain	ISX_1	0.003	0.187	0.012
14	C/T	HOXA4	gain	HOXA4_1	0.003	0.118	0.003
15	C/T	NFIB	gain	NFIB_1	0.003	0.074	0.009
16	C/T	BARHL1	gain	BARHL1_3	0.003	0.309	0.005
17	C/T	GBX1	gain	GBX1_1	0.003	0.157	0.007
18	C/T	GSX2	gain	GSX2_1	0.004	0.290	0.017
19	C/T	T	gain	MA0009.1	0.004	0.233	0.018
20	C/T	VAX1	gain	VAX1_2	0.004	0.271	0.011
21	C/T	Nobox	gain	MA0125.1	0.004	0.190	0.009
22	C/T	LHX2	gain	LHX2_1	0.004	0.360	0.027
23	C/T	HOXB3	gain	HOXB3_1	0.005	0.454	0.027
24	C/T	HOXA5	gain	HOXA5_3	0.005	0.301	0.024
25	C/T	DLX2	gain	DLX2_1	0.006	0.098	0.005
26	C/T	BARHL2	gain	BARHL2_3	0.006	0.265	0.008
27	C/T	GBX2	gain	GBX2_1	0.006	0.182	0.014
28	C/T	SOX21	gain	SOX21_4	0.007	0.468	0.023
29	C/T	HOXA5	gain	HOXA5_2	0.007	0.409	0.022
30	C/T	SHOX2	gain	SHOX2_2	0.007	0.140	0.009
31	C/T	EN2	gain	EN2_1	0.007	0.328	0.02599
32	C/T	NKX1-2	gain	NKX1-2_1	0.007	0.568	0.0367
33	C/T	VSX1	gain	VSX1_2	0.007	0.109	0.007
34	C/T	ISX	gain	ISX_4	0.007	0.328	0.010
35	C/T	HOXA1	gain	HOXA1_1	0.008	0.257	0.027
36	C/T	NR1H4	gain	NR1H4_1	0.008	0.102	0.015
37	C/T	VAX1	gain	VAX1_1	0.009	0.391	0.030
38	C/T	HOXA6	gain	HOXA6_1	0.009	0.521	0.037
39	C/T	HOXA3	gain	HOXA3_2	0.009	0.395	0.028
40	C/T	POU3F2	gain	POU3F2_3	0.010	0.272	0.041
41	C/T	EMX2	gain	EMX2_2	0.010	0.202	0.011
42	C/T	ALX3	gain	ALX3_2	0.010	0.110	0.010
43	C/T	NKX1-1	gain	NKX1-1_1	0.010	0.553	0.042
44	C/T	HOXA2	gain	HOXA2_1	0.010	0.251	0.023
45	C/T	MSX2	gain	PROP1_2	0.0001	0.769	0.002
46	C/T	PRDM1	gain	MA0132.1	0.0002	0.143	0.007
47	C/T	HOXB5	gain	VSX2_1	0.0004	0.175	0.006
48	C/T	POU2F1	gain	NOBOX_1	0.001	0.694	0.008
49	C/T	VSX2	gain	MEIS1::HOXA9_2	0.001	0.089	0.006
50	C/T	SREBF	gain	SOX10_1	0.002	0.323	0.010
51	C/T	HESX1	gain	BARHL2_6	0.003	0.375	0.004
52	C/T	SOX3	gain	DLX5_1	0.003	0.330	0.024
53	C/T	VSX1	gain	PDX1_3	0.003	0.200	0.004

54	C/T	EVX1	gain	NKX2-1_3	0.003	0.552	0.021
55	C/T	PDX1	gain	TGIF1_1	0.003	0.054	0.002
56	C/T	DLX3	gain	NKX2-6_1	0.003	0.293	0.011
57	C/T	PBX1	gain	ISX_1	0.003	0.187	0.012
58	C/T	GATA1	gain	HOXA4_1	0.003	0.118	0.003
59	C/T	NKX6-1	gain	NFIB_1	0.003	0.074	0.009
60	C/T	LBX2	gain	BARHL1_3	0.003	0.309	0.005
61	C/T	Myb	gain	GBX1_1	0.003	0.157	0.007
62	C/T	HOXD3	gain	GSX2_1	0.004	0.290	0.017
63	C/T	NFIA	gain	MA0009.1	0.004	0.233	0.018
64	C/T	VAX2	gain	VAX1_2	0.004	0.271	0.011
65	C/T	RAX	gain	MA0125.1	0.004	0.190	0.009
66	C/T	TFAP4	gain	LHX2_1	0.004	0.360	0.027
67	C/T	DLX1	gain	HOXB3_1	0.005	0.454	0.027
68	C/T	CTCF	gain	HOXA5_3	0.005	0.301	0.024
69	C/T	DLX5	gain	DLX2_1	0.006	0.098	0.005
70	C/T	HOXA5	gain	BARHL2_3	0.006	0.265	0.008
71	C/T	MSX1	gain	GBX2_1	0.006	0.182	0.014
72	C/T	SOX7	gain	SOX21_4	0.007	0.468	0.023
73	C/T	EP300	gain	HOXA5_2	0.007	0.409	0.022
74	C/T	PRRX2	gain	SHOX2_2	0.007	0.140	0.009
75	C/T	ALX3	gain	EN2_1	0.007	0.328	0.026
76	C/T	PDX1	gain	NKX1-2_1	0.007	0.568	0.037
77	C/T	DLX1	gain	VSX1_2	0.007	0.109	0.007
78	C/T	VSX1	gain	ISX_4	0.007	0.328	0.010
79	C/T	SOX5	gain	HOXA1_1	0.008	0.257	0.027
80	C/T	SOX13	gain	NR1H4_1	0.008	0.102	0.015
81	C/T	FOXM1	gain	VAX1_1	0.009	0.391	0.030
82	C/T	T	gain	HOXA6_1	0.009	0.521	0.037
83	C/T	SMAD3	gain	HOXA3_2	0.009	0.395	0.028
84	C/T	HOXA3	gain	POU3F2_3	0.010	0.272	0.041
85	C/T	GATA2	gain	EMX2_2	0.010	0.202	0.011
86	C/T	DLX3	gain	ALX3_2	0.010	0.110	0.010
87	C/T	LHX2	gain	NKX1-1_1	0.010	0.553	0.042
88	C/T	Gata4	gain	HOXA2_1	0.010	0.251	0.023
89	C/T	MNX1	gain	PROP1_2	0.000	0.769	0.002
90	C/T	PRRX2	gain	MA0132.1	0.000	0.143	0.007
91	C/T	MYB	gain	VSX2_1	0.000	0.175	0.006
92	C/T	NKX2-5	gain	NOBOX_1	0.001	0.694	0.008
93	C/T	EVX2	gain	MEIS1::HOXA9_2	0.001	0.089	0.006
94	C/T	BSX	gain	SOX10_1	0.002	0.323	0.010
95	C/T	EVX2	gain	BARHL2_6	0.003	0.375	0.004
96	C/T	LHX9	gain	DLX5_1	0.003	0.330	0.024
97	C/T	HOXB3	gain	PDX1_3	0.003	0.200	0.004
98	C/T	GBX2	gain	NKX2-1_3	0.003	0.552	0.021

99	C/T	SMAD2::SMAD3::SMAD4	gain	TGIF1_1	0.003	0.054	0.002
100	C/T	LBX2	gain	NKX2-6_1	0.003	0.293	0.011
101	C/T	SHOX2	gain	ISX_1	0.003	0.187	0.012
102	C/T	EVX1	gain	HOXA4_1	0.003	0.118	0.003
103	C/T	MSX2	gain	NFIB_1	0.003	0.074	0.009
104	C/T	DLX2	gain	BARHL1_3	0.003	0.309	0.005
105	C/T	UNCX	gain	GBX1_1	0.003	0.157	0.007
106	C/T	GBX1	gain	GSX2_1	0.004	0.290	0.017
107	C/T	VSX1	gain	MA0009.1	0.004	0.233	0.018
108	C/T	HMX1	gain	VAX1_2	0.004	0.271	0.011
109	C/T	Prrx2	gain	MA0125.1	0.004	0.190	0.009
110	C/T	DLX4	gain	LHX2_1	0.004	0.360	0.027
111	C/T	HESX1	gain	HOXB3_1	0.005	0.454	0.027
112	C/T	NFY	gain	HOXA5_3	0.005	0.301	0.024
113	C/T	PAX7	gain	DLX2_1	0.006	0.098	0.005
114	C/T	SIRT6	gain	BARHL2_3	0.006	0.265	0.008
115	C/T	SHOX	gain	GBX2_1	0.006	0.182	0.014
116	C/T	RUNX1	gain	SOX21_4	0.007	0.468	0.023
117	C/T	HOXB5	gain	HOXA5_2	0.007	0.409	0.022
118	C/T	GBX2	gain	SHOX2_2	0.007	0.140	0.009
119	C/T	LMX1B	gain	EN2_1	0.007	0.328	0.026
120	C/T	HOXB2	gain	NKX1-2_1	0.007	0.568	0.037
121	C/T	PDX1	gain	VSX1_2	0.007	0.109	0.007
122	C/T	GSX1	gain	ISX_4	0.007	0.328	0.010
123	C/T	HOXA2	gain	HOXA1_1	0.008	0.257	0.027
124	C/T	NKX3-2	gain	NR1H4_1	0.008	0.102	0.015
125	C/T	RAD21	gain	VAX1_1	0.009	0.391	0.030
126	C/T	PDX1	gain	HOXA6_1	0.009	0.521	0.037
127	C/T	TBX1	gain	HOXA3_2	0.009	0.395	0.028
128	C/T	GATA	loss	GATA_disc4	0.003	0.003	0.054
129	C/T	GATA3	loss	GATA3_2	0.004	0.016	0.932
130	C/T	HDX	loss	HDX_1	0.005	0.007	0.130
131	C/T	PBX1	loss	PBX1_4	0.006	0.015	0.308
132	C/T	HDAC2	loss	HDAC2_disc3	0.006	0.008	0.122
133	C/T	PBX3	loss	PBX3_disc2	0.009	0.025	0.315
134	C/T	HOXD3	loss	GATA_disc4	0.003	0.003	0.054
135	C/T	BARHL1	loss	GATA3_2	0.004	0.016	0.932
136	C/T	RAX	loss	HDX_1	0.005	0.007	0.130
137	C/T	MEOX1	loss	PBX1_4	0.006	0.015	0.308
138	C/T	SOX1	loss	HDAC2_disc3	0.006	0.008	0.122
139	C/T	GBX2	loss	PBX3_disc2	0.009	0.025	0.315
140	C/T	RUNX1	loss	GATA_disc4	0.003	0.003	0.054
141	C/T	SOX17	loss	GATA3_2	0.004	0.016	0.932

142	C/T	GSX2	loss	HDX_1	0.005	0.007	0.130
143	C/T	MSX1	loss	PBX1_4	0.006	0.015	0.308
144	C/T	GBX1	loss	HDAC2_disc 3	0.006	0.008	0.122
145	C/T	HMX3	loss	PBX3_disc2	0.009	0.025	0.315
	T[8]	Cerebral cortex GABAergic interneuron fate commitment (GO:0021892; FDR = **0.006**);<br>Neurogenesis (GO:0022008; FDR = **2.23×10^{-15}**);<br>Regulation of cell death (GO:0010941; FDR = **0.0001**);<br>Cardiac muscle tissue regeneration (GO:0061026; FDR = **0.01**);<br>Forebrain dorsal/ventral pattern formation (GO:0021798; FDR = **0.03**);<br>BMP signaling pathway involved in heart development (GO:0061312; FDR = **0.03**);<br>Cellular response to growth factor stimulus (GO:0071363; FDR = **0.04**);<br>Forebrain neuron fate commitment (GO:0021877; FDR = **0.03**);<br>Hypothalamus development (GO:0021854; FDR = **8.76×10^{-9}**);<br>Neuroblast differentiation (GO:0014016; FDR = **0.03**);<br>Forebrain morphogenesis (GO:0048853; FDR = **0.002**);<br>Cardiac pacemaker cell development (GO:0060926; FDR = **0.04**);<br>Negative regulation of oligodendrocyte differentiation (GO:0048715; FDR = **0.002**);<br>Negative regulation of neurogenesis (GO:0050768; FDR = **0.003**);<br>Ventricular cardiac muscle cell development (GO:0055015; FDR = **0.04**);<br>Cardiac atrium morphogenesis (GO:0003209; FDR = **0.02**);<br>Neuron fate specification (GO:0048665; FDR = **0.02**);<br>Oligodendrocyte differentiation (GO:0048709; FDR = **0.0001**);<br>Neural precursor cell proliferation (GO:0061351; FDR = **3.57×10^{-5}**);<br>Heart valve development (GO:0003170; FDR = **1.02×10^{-2}**);<br>Neuron migration (GO:0001764; FDR = **2.17×10^{-5}**);<br>Hippocampus development (GO:0021766; FDR = **2.27×10^{-5}**);<br>Regulation of neural precursor cell proliferation (GO:2000177; FDR = **3.18×10^{-2}**);<br>Blood vessel morphogenesis (GO:0048514; FDR = **4.65×10^{-3}**).					
	C[9]	Forebrain neuron development (GO:0021884; FDR = **0.045**);<br>Neuron fate commitment (GO:0048663; FDR = **0.009**);<br>Neuron migration (GO:0001764; FDR = **0.048**);<br>Regulation of protein stability (GO:0031647; FDR = **0.04**).					

1 – Reference (Ref) / alternative (SNP) allele;
2 – TF - transcription factor;
3 – Binding of TF to the reference (LOSS) / alternative (GAIN) allele;
4 – Binding sites with high affinity for TF;
5 – *p*-value statistically confirming the potential gain or loss of function of the genomic region with SNP in terms of transcription factor binding;
6 – *p*-value for assessing the binding of TF to the Ref allele;
7 – *p*-value for assessing the binding of TF to the SNP allele;
8 – Biological processes pathogenetically significant for IS, in which TFs that bind to the SNP allele are jointly involved (data from the Gene Ontology resource; http://geneontology.org/);
9 – Biological processes pathogenetically significant for IS, in which TFs that bind to reference allele are jointly involved (data from the Gene Ontology resource; http://geneontology.org/).

Table S6: Analysis of the effect of rs12561767 *SERBP1* on the binding of DNA to transcription factors.

№	Ref/ SNP allele[1]	TF[2]	GAIN /LOSS[3]	Motif[4]	*p*-Value SNP impact[5]	*p*-Value Ref[6]	*p*-Value SNP[7]
1	G/A	NFIC	gain	NFIC_2	0.0002	0.052	0.00001
2	G/A	POU1F1	gain	POU1F1_2	0.0004	0.226	0.007
3	G/A	TCF7	gain	TCF7_1	0.001	0.166	0.012
4	G/A	LEF1	gain	LEF1_2	0.001	0.455	0.044
5	G/A	SP1	gain	SP1_disc2	0.002	0.216	0.014
6	G/A	HOXA13	gain	HOXA13_5	0.003	0.359	0.022
7	G/A	CCDC6	gain	CCDC6_1	0.003	0.301	0.016
8	G/A	MEF2D	gain	MEF2D_1	0.004	0.444	0.024
9	G/A	YY1	gain	YY1_3	0.005	0.054	0.004
10	G/A	RAD21	gain	RAD21_disc3	0.005	0.158	0.014
11	G/A	BARHL2	gain	BARHL2_5	0.007	0.604	0.042
12	G/A	MYEF2	gain	MYEF2_7	0.007	0.181	0.014
13	G/A	PBX1	gain	PBX1_5	0.008	0.252	0.025
14	G/A	TBP	gain	TBP_1	0.008	0.198	0.025
15	G/A	RXRB	gain	RXRB_2	0.009	0.309	0.044
16	G/A	THAP1	gain	THAP1_disc2	0.010	0.083	0.010
17	G/A	NR2C2	gain	MA0504.1	0.010	0.324	0.029
18	G/A	LEF1	gain	LEF1_4	0.010	0.244	0.024
19	G/A	PDX1	gain	PDX1_2	0.011	0.267	0.028
20	G/A	ARID5A	gain	ARID5A_1	0.011	0.345	0.036
21	G/A	TCF7	gain	TCF7_2	0.012	0.069	0.006
22	G/A	BARHL1	gain	BARHL1_2	0.013	0.642	0.037
23	G/A	TCF7L2	gain	TCF7L2_2	0.013	0.231	0.028
24	G/A	RXRG	gain	RXRG_1	0.014	0.052	0.006
25	G/A	TCF7L2	gain	TCF7L2_disc2	0.014	0.159	0.015
26	G/A	NANOG	gain	NANOG_disc 4	0.016	0.300	0.044
27	G/A	MEF2C	gain	MA0497.1	0.017	0.078	0.010
28	G/A	RXRB	gain	RXRB_1	0.019	0.069	0.009
29	G/A	RXRA	gain	RXRA_3	0.020	0.227	0.046
30	G/A	RXRA	gain	RXRA_10	0.022	0.118	0.017
31	G/A	LEF1	gain	LEF1_5	0.023	0.109	0.018
32	G/A	BARHL2	gain	BARHL2_2	0.027	0.375	0.038
33	G/A	YY1	gain	YY1_1	0.028	0.124	0.019
34	G/A	SOX7	gain	SOX7_2	0.030	0.152	0.025
35	G/A	HESX1	gain	HESX1_2	0.032	0.210	0.042
36	G/A	ELK1	gain	NFIC_2	0.0002	0.052	0.00001
37	G/A	RXRA	gain	POU1F1_2	0.0004	0.226	0.007
38	G/A	NR4A2	gain	TCF7_1	0.001	0.166	0.012
39	G/A	AP3	loss	AP3_1	0.007	0.026	0.437
40	G/A	SOX10	loss	SOX10_2	0.007	0.020	0.296
41	G/A	HSF1	loss	HSF1_1	0.008	0.026	0.594
42	G/A	HOXA5	loss	HOXA5_2	0.009	0.020	0.375
43	G/A	PBX1	loss	PBX1_4	0.010	0.045	0.415

44	G/A	HNF4	loss	HNF4_disc2	0.011	0.011	0.108
45	G/A	PAX	loss	PAX_1	0.014	0.037	0.664
46	G/A	HINFP	loss	MA0131.1	0.015	0.028	0.343
47	G/A	ALX4	loss	ALX4_1	0.016	0.034	0.465
48	G/A	TOPORS	loss	TOPORS_1	0.019	0.050	0.240
49	G/A	HOXA7	loss	HOXA7_1	0.019	0.022	0.219
50	G/A	ATF3	loss	ATF3_disc2	0.025	0.012	0.072
51	G/A	TEAD1	loss	TEAD1_3	0.029	0.038	0.307
52	G/A	ELK1	loss	MA0028.1	0.032	0.043	0.352
53	G/A	REST	loss	REST_2	0.033	0.029	0.163
	A[8]	Steroid hormone mediated signaling pathway (GO:0043401; FDR=**0.038**); Neuron migration (GO:0001764; FDR=**0.01**); Apoptotic process (GO:0006915; FDR=**0.03**); Nervous system development (GO:0007399; FDR=**0.004**); Generation of neurons (GO:0048699; FDR=**0.04**); Programmed cell death (GO:0012501; FDR=**0.03**); Cell death (GO:0008219; FDR=**0.04**).					
	G[9]	Regulation of apoptotic process (GO:0042981; FDR=**0.008**). regulation of programmed cell death (GO:0043067; FDR=**0.009**)					

1 – Reference (Ref) / alternative (SNP) allele;
2 – TF - transcription factor;
3 – Binding of TF to the reference (LOSS) / alternative (GAIN) allele;
4 – Binding sites with high affinity for TF;
5 – *p*-value statistically confirming the potential gain or loss of function of the genomic region with SNP in terms of transcription factor binding;
6 – *p*-value for assessing the binding of TF to the Ref allele;
7 – *p*-value for assessing the binding of TF to the SNP allele;
8 – Biological processes pathogenetically significant for IS, in which TFs that bind to the SNP allele are jointly involved (data from the Gene Ontology resource; http://geneontology,org/);
9 – Biological processes pathogenetically significant for IS, in which TFs that bind to reference allele are jointly involved (data from the Gene Ontology resource; http://geneontology,org/).

Table S7: Analysis of the effect of rs12566098 *SERBP1* on the binding of DNA to transcription factors.

№	Ref/ SNP allele[1]	TF[2]	GAIN /LOSS[3]	Motif[4]	*p*-Value SNP impact[5]	*p*-Value Ref[6]	*p*-Value SNP[7]
1	C/G	DBP	gain	DBP_1	0.0003	0.223	0.007
2	C/G	RXRA	gain	RXRA_disc5	0.003	0.147	0.012
3	C/G	GFI1	gain	GFI1_3	0.006	0.371	0.022
4	C/G	MYEF2	gain	MYEF2_7	0.007	0.204	0.044
5	C/G	RAD21	gain	RAD21_disc 3	0.008	0.083	0.010
6	C/G	NKX2-3	gain	NKX2-3_1	0.010	0.078	0.007
7	C/G	SOX1	gain	SOX1_4	0.013	0.154	0.016
8	C/G	FOXO1	gain	DBP_1	0.0003	0.223	0.007
9	C/G	SOX5	gain	RXRA_disc5	0.003	0.147	0.012

10	C/G	FOXL1	loss	FOXL1_1	0.000001	0.0000002	0.066
11	C/G	HNF1	loss	HNF1_3	0.000004	0.00003	0.100
12	C/G	FOXO4	loss	FOXO4_2	0.0004	0.006	0.110
13	C/G	TBX21	loss	TBX21_6	0.001	0.021	0.799
14	C/G	RUNX2	loss	RUNX2_1	0.001	0.007	0.595
15	C/G	RUNX	loss	RUNX_2	0.001	0.012	0.348
16	C/G	GATA4	loss	GATA4_1	0.002	0.009	0.117
17	C/G	HMBOX1	loss	HMBOX1_1	0.002	0.020	0.426
18	C/G	FOXQ1	loss	FOXQ1_1	0.002	0.008	0.148
19	C/G	RUNX3	loss	RUNX3_4	0.002	0.011	0.783
20	C/G	RUNX3	loss	RUNX3_2	0.002	0.013	0.749
21	C/G	NFY	loss	NFY_5	0.002	0.023	0.503
22	C/G	HNF1	loss	HNF1_1	0.002	0.006	0.184
23	C/G	FOXF2	loss	MA0030.1	0.002	0.004	0.081
24	C/G	ZNF652	loss	ZNF652_1	0.003	0.036	0.698
25	C/G	ZNF354C	loss	MA0130.1	0.003	0.010	0.220
26	C/G	RUNX2	loss	RUNX2_6	0.003	0.007	0.193
27	C/G	RUNX3	loss	RUNX3_3	0.004	0.005	0.063
28	C/G	OBOX5	loss	OBOX5_2	0.004	0.021	0.305
29	C/G	IRF5	loss	IRF5_2	0.004	0.035	0.999
30	C/G	FOXF2	loss	FOXF2_1	0.005	0.009	0.160
31	C/G	SMARC	loss	SMARC_disc2	0.005	0.032	0.366
32	C/G	OBOX5	loss	OBOX5_1	0.005	0.034	0.443
33	C/G	FOXF2	loss	FOXF2_2	0.005	0.004	0.095
34	C/G	FOXO1	loss	FOXO1_2	0.005	0.024	0.334
35	C/G	NFY	loss	NFY_1	0.006	0.009	0.179
36	C/G	HNF1A	loss	MA0046.1	0.006	0.017	0.155
37	C/G	Foxq1	loss	MA0040.1	0.006	0.009	0.125
38	C/G	FOXJ3	loss	FOXJ3_7	0.006	0.019	0.534
39	C/G	FOXO3	loss	FOXO3_5	0.006	0.007	0.257
40	C/G	RUNX2	loss	RUNX2_5	0.008	0.010	0.113
41	C/G	FOXH1	loss	MA0479.1	0.008	0.042	0.325
42	C/G	GRHL1	loss	GRHL1_3	0.008	0.041	0.932
43	C/G	FOXO3	loss	FOXO3_2	0.010	0.028	0.306
44	C/G	ZNF354C	loss	ZNF354C_1	0.010	0.007	0.222
45	C/G	FOXQ1	loss	FOXQ1_2	0.010	0.018	0.224
46	C/G	CREB1	loss	CREB1_3	0.010	0.011	0.177
47	C/G	AFP	loss	AFP_1	0.010	0.013	0.145
48	C/G	MYF6	loss	MYF6_2	0.011	0.018	0.476
49	C/G	FOXL1	loss	FOXL1_4	0.011	0.013	0.339
50	C/G	GLI2	loss	GLI2_2	0.012	0.039	0.395
51	C/G	ZNF524	loss	ZNF524_1	0.013	0.031	0.365
52	C/G	TFCP2	loss	TFCP2_4	0.013	0.019	0.227
53	C/G	PAX4	loss	FOXL1_1	0.000001	0.0000002	0.066
54	C/G	ZBTB14	loss	HNF1_3	0.000004	0.00003	0.100
55	C/G	RUNX3	loss	FOXO4_2	0.0004	0.006	0.110
56	C/G	ZBTB6	loss	TBX21_6	0.001	0.021	0.799
57	C/G	RUNX1	loss	RUNX2_1	0.001	0.007	0.595
58	C/G	SIX5	loss	RUNX_2	0.001	0.012	0.348

59	C/G	SRY	loss	GATA4_1	0.002	0.009	0.117
60	C/G	ZBTB7B	loss	HMBOX1_1	0.002	0.020	0.426
61	C/G	POU2F1	loss	FOXQ1_1	0.002	0.008	0.148
62	C/G	FOXO4	loss	RUNX3_4	0.002	0.011	0.783
63	C/G	FOXO1	loss	RUNX3_2	0.002	0.013	0.749
64	C/G	GFI1	loss	NFY_5	0.002	0.023	0.503
65	C/G	EGR1	loss	HNF1_1	0.002	0.006	0.184
66	C/G	KLF12	loss	MA0030.1	0.002	0.004	0.081
67	C/G	ARID3A	loss	ZNF652_1	0.003	0.036	0.698
68	C/G	OBOX6	loss	MA0130.1	0.003	0.010	0.220
69	C/G	RHOXF1	loss	RUNX2_6	0.003	0.007	0.193
70	C/G	SOX5	loss	RUNX3_3	0.004	0.005	0.063
71	C/G	FOXI1	loss	OBOX5_2	0.004	0.021	0.305
	G[8]	-					
	C[9]	Cellular response to growth factor stimulus (GO:0071363; FDR = **0.03**); Positive regulation of cytokine production (GO:0001819; FDR = **0.03**).					

1 – Reference (Ref) / alternative (SNP) allele;
2 – TF - transcription factor;
3 – Binding of TF to the reference (LOSS) / alternative (GAIN) allele;
4 – Binding sites with high affinity for TF;
5 – *p*-value statistically confirming the potential gain or loss of function of the genomic region with SNP in terms of transcription factor binding;
6 – *p*-value for assessing the binding of TF to the Ref allele;
7 – *p*-value for assessing the binding of TF to the SNP allele;
8 – Biological processes pathogenetically significant for IS, in which TFs that bind to the alternative allele are jointly involved (data from the Gene Ontology resource; http://geneontology,org/);
9 – Biological processes pathogenetically significant for AI, in which TFs that bind to an reference allele are jointly involved (data from the Gene Ontology resource; http://geneontology,org/).

Table S8: Analysis of the effect of rs6702742 *SERBP1* on the binding of DNA to transcription factors

№	Ref/ SNP allele[1]	TF[2]	GAIN /LOSS[3]	Motif[4]	*p*-Value SNP impact[5]	*p*-Value Ref[6]	*p*-Value SNP[7]
1	A/G	TFAP2A	gain	TFAP2A_4	0	0.160	0.000003
2	A/G	TFAP2A	gain	TFAP2A_1	0	0.160	0.000003
3	A/G	TFAP2C	gain	TFAP2C_1	0.0004	0.194	0.007
4	A/G	GLIS3	gain	GLIS3_1	0.001	0.093	0.004
5	A/G	TFAP2	gain	TFAP2_3	0.002	0.398	0.022
6	A/G	CTCF	gain	CTCF_disc8	0.005	0.201	0.035
7	A/G	HIC1	gain	HIC1_4	0.006	0.200	0.023
8	A/G	TCF12	gain	TCF12_disc3	0.007	0.153	0.012
9	A/G	REST	gain	REST_disc3	0.008	0.552	0.049
10	A/G	TP53	gain	TP53_2	0.008	0.465	0.025
11	A/G	GATA	gain	GATA_disc5	0.009	0.221	0.028
12	A/G	E2F1	gain	E2F1_14	0.010	0.305	0.038

13	A/G	E2F1	gain	E2F1_19	0.011	0.239	0.026
14	A/G	TP53	gain	TP53_3	0.013	0.221	0.017
15	A/G	E2F1	gain	E2F1_20	0.016	0.315	0.048
16	A/G	TFAP2C	gain	TFAP2C_4	0.021	0.082	0.017
17	A/G	SMAD	gain	SMAD_2	0.022	0.357	0.041
18	A/G	ESRRA	gain	TFAP2A_4	0	0.160	0.000003
19	A/G	TFAP2C	gain	TFAP2A_1	0	0.160	0.000003
20	A/G	CREB1	gain	TFAP2C_1	0.0004	0.194	0.007
21	A/G	OBOX5	gain	GLIS3_1	0.001	0.093	0.004
22	A/G	CUX1	gain	TFAP2_3	0.002	0.398	0.022
23	A/G	Pax4	gain	CTCF_disc8	0.005	0.201	0.035
24	A/G	NR1H4	loss	NR1H4_3	0	0.000002	0.092
25	A/G	PAX5	loss	PAX5_2	0.0004	0.048	0.240
26	A/G	HNF4A	loss	HNF4A_8	0.001	0.012	0.286
27	A/G	MYBL1	loss	MYBL1_2	0.002	0.009	0.486
28	A/G	HNF1	loss	HNF1_4	0.002	0.014	0.177
29	A/G	ZBTB7A	loss	ZBTB7A_1	0.002	0.026	0.307
30	A/G	RXRA	loss	RXRA_5	0.002	0.008	0.253
31	A/G	HNF1A	loss	MA0046.1	0.003	0.007	0.087
32	A/G	MYBL2	loss	MYBL2_4	0.006	0.008	0.100
33	A/G	REL	loss	REL_2	0.006	0.019	0.585
34	A/G	HDAC2	loss	HDAC2_disc 5	0.006	0.047	0.356
35	A/G	RUNX2	loss	RUNX2_6	0.006	0.007	0.198
36	A/G	ATF4	loss	ATF4_1	0.006	0.019	0.155
37	A/G	AR	loss	AR_6	0.007	0.038	0.529
38	A/G	REL	loss	REL_1	0.007	0.016	0.477
39	A/G	MYB	loss	MYB_2	0.007	0.041	0.774
40	A/G	SOX9	loss	SOX9_9	0.008	0.044	0.406
41	A/G	HNF1A	loss	HNF1A_2	0.010	0.036	0.372
42	A/G	NR2F1	loss	NR2F1_5	0.010	0.046	0.462
43	A/G	NR2F1	loss	NR2F1_4	0.011	0.003	0.111
44	A/G	TATA	loss	TATA_disc2	0.012	0.026	0.315
45	A/G	HNF1	loss	HNF1_3	0.014	0.015	0.115
46	A/G	ETS	loss	ETS_disc4	0.016	0.031	0.396
47	A/G	VDR	loss	VDR_2	0.017	0.017	0.182
48	A/G	HNF1A	loss	HNF1A_3	0.017	0.031	0.238
49	A/G	ZBTB7A	loss	ZBTB7A_dis c1	0.017	0.034	0.193
50	A/G	ZNF652	loss	ZNF652_1	0.017	0.006	0.058
51	A/G	OBOX5	loss	OBOX5_2	0.019	0.013	0.102
52	A/G	ATF2	loss	ATF2_3	0.021	0.014	0.211
53	A/G	ESRRB	loss	ESRRB_1	0.021	0.033	0.237
54	A/G	MYBL1	loss	MYBL1_1	0.022	0.026	0.314
55	A/G	RARA	loss	RARA_1	0.022	0.049	0.372
56	A/G	ZBTB33	loss	ZBTB33_dis c4	0.023	0.006	0.134

57	A/G	AR	loss	NR1H4_3	0	0.000002	0.092
58	A/G	TFAP2A	loss	PAX5_2	0.0004	0.048	0.240
59	A/G	LHX9	loss	HNF4A_8	0.001	0.012	0.286
60	A/G	MYBL1	loss	MYBL1_2	0.002	0.009	0.486
61	A/G	ESRRG	loss	HNF1_4	0.002	0.014	0.177
62	A/G	SMC3	loss	ZBTB7A_1	0.002	0.026	0.307
63	A/G	TFAP2B	loss	RXRA_5	0.002	0.008	0.253
64	A/G	CTCF	loss	MA0046.1	0.003	0.007	0.087
	G[8]	Positive regulation of neuron death (GO:1901216; FDR = **0.01**); Regulation of neurogenesis (GO:0050767; FDR = **0.03**).					
	A[9]	Positive regulation of neuron apoptotic process (GO:0043525; FDR = $\mathbf{3.88\times10^{-6}}$); Positive regulation of cytokine production (GO:0001819; FDR = **0.03**); Negative regulation of apoptotic process (GO:0043066; FDR = **0.002**).					

1 – Reference (Ref) / alternative (SNP) allele;
2 – TF - transcription factor;
3 – Binding of TF to the reference (LOSS) / alternative (GAIN) allele;
4 – Binding sites with high affinity for TF;
5 – *p*-value statistically confirming the potential gain or loss of function of the genomic region with SNP in terms of transcription factor binding;
6 – *p*-value for assessing the binding of TF to the Ref allele;
7 – *p*-value for assessing the binding of TF to the SNP allele;
8 – Biological processes pathogenetically significant for IS, in which TFs that bind to the alternative allele are jointly involved (data from the Gene Ontology resource; http://geneontology,org/);
9 – Biological processes pathogenetically significant for AI, in which TFs that bind to an reference allele are jointly involved (data from the Gene Ontology resource; http://geneontology,org/).

Table S9: Main functional characteristics of predicted functional partners of *SERBP1* (STRING database).

Protein	Functions	*			Score
		1	2	3	
PGRMC1	Membrane-associated progesterone receptor component 1; Component of a progesterone-binding protein complex; binds progesterone; has many reported cellular functions (heme homeostasis, interaction with CYPs); belongs to the cytochrome b5 family. MAPR subfamily.			✓	0.986
RPS29	Small subunit ribosomal protein s29e; ribosomal protein S29. This gene encodes a ribosomal protein that is a component of the 40S subunit and a member of the S14P family of ribosomal proteins.	✓	✓	✓	0.984
RPS24	40S ribosomal protein S24; required for processing of pre-rRNA and maturation of 40S ribosomal subunits; belongs to the eukaryotic ribosomal protein eS24 family	✓	✓		0.983
RPL21	60S ribosomal protein L21; component of the large ribosomal subunit	✓	✓	✓	0.982

RPL5	60S ribosomal protein L5; component of the ribosome, a large ribonucleoprotein complex responsible for the synthesis of proteins in the cell. The small ribosomal subunit (SSU) binds messenger RNAs (mRNAs) and translates the encoded message by selecting cognate aminoacyl-transfer RNA (tRNA) molecules. The large subunit (LSU) contains the ribosomal catalytic site termed the peptidyl transferase center (PTC), which catalyzes the formation of peptide bonds, thereby polymerizing the amino acids delivered by tRNAs into a polypeptide chain. The nascent polypeptides leave the ribosome through a tunnel in the LSU and interact with protein factors that function in enzymatic processing, targeting, and the membrane insertion of nascent chains at the exit of the ribosomal tunnel. As part of the 5S RNP/5S ribonucleoprotein particle it is an essential component of the LSU, required for its formation and the maturation of rRNAs). It also couples ribosome biogenesis to p53/TP53 activation. As part of the 5S RNP, it accumulates in the nucleoplasm and inhibits MDM2 when ribosome biogenesis is perturbed, mediating the stabilization and the activation of TP53.	✓	✓	✓	0.981
RPS6	40S ribosomal protein S6; may play an important role in controlling cell growth and proliferation through the selective translation of particular classes of mRNA. It is the major substrate of protein kinases in the ribosome, with subsets of five C-terminal serine residues phosphorylated by different protein kinases. Phosphorylation is induced by a wide range of stimuli, including growth factors, tumor-promoting agents, and mitogens. Dephosphorylation occurs at growth arrest. The protein may contribute to the control of cell growth and proliferation through the selective translation of particular classes of mRNA.	✓	✓	✓	0.981
RPL23	Large subunit ribosomal protein l23e; ribosomal protein L23. This gene encodes a ribosomal protein that is a component of the 60S subunit. The protein belongs to the L14P family of ribosomal proteins. It is located in the cytoplasm. This gene has been referred to as rpL17 because the encoded protein shares amino acid identity with ribosomal protein L17 from Saccharomyces cerevisiae; however, its official symbol is RPL23.	✓	✓	✓	0.981
RPS25	Small subunit ribosomal protein s25e; ribosomal protein S25. This gene encodes a ribosomal protein that is a component of the 40S subunit. The protein belongs to the S25E family of ribosomal proteins. It is located in the cytoplasm.	✓	✓	✓	0.980
RPL8	60S ribosomal protein L8; component of the large ribosomal subunit. This gene encodes a ribosomal protein that is a component of the 60S subunit. The protein belongs to the L2P family of ribosomal proteins. It is located in the cytoplasm. In rat, the protein associates with the 5.8S rRNA. It very likely participates in the binding of aminoacyl-tRNA and is a constituent of the elongation factor 2-binding site at the ribosomal subunit interface. Alternatively spliced transcript variants encoding the same protein exist.	✓	✓	✓	0.979

RPS19	40S ribosomal protein S19; required for pre-rRNA processing and maturation of 40S ribosomal subunits; belongs to the eukaryotic ribosomal protein eS19 family. It is located in the cytoplasm.	✓	✓		0.979
* - Proven interaction mechanisms:<br>1 – Coexpression; 2 – Experiments; 3 - Textmining					

Table S10: Functional enrichments of *SERBP1* network

№	Term ID	Term Description	Observed Gene Count	Background Gene Count	Strength	FDR
	Biological processes (Gene Ontology)					
1	GO:0006614	SRP-dependent cotranslational protein targeting to membrane	9	96	2.22	1.81×10^{-15}
2	GO:0000184	Nuclear-transcribed mrna catabolic process, nonsense-mediated decay	9	119	2.13	1.98×10^{-15}
3	GO:0019083	Viral transcription	9	115	2.14	1.98×10^{-15}
4	GO:0006413	Translational initiation	9	141	2.06	5.85×10^{-15}
5	GO:1901566	Organonitrogen compound biosynthetic process	10	1346	1.12	1.05×10^{-8}
6	GO:0044271	Cellular nitrogen compound biosynthetic process	10	1522	1.07	3.24×10^{-8}
7	GO:0051649	Establishment of localization in cell	10	2375	0.87	2.12×10^{-6}
8	GO:0046483	Heterocycle metabolic process	10	2840	0.8	9.41×10^{-6}
9	GO:0006725	Cellular aromatic compound metabolic process	10	2882	0.79	1.07×10^{-5}
10	GO:1901360	Organic cyclic compound metabolic process	10	3118	0.76	2.12×10^{-5}
11	GO:0006810	Transport	10	4353	0.61	4.9×10^{-4}
12	GO:0006364	rRNA processing	4	212	1.53	0.0008
13	GO:0010468	Regulation of gene expression	10	4813	0.57	0.0012
14	GO:0042274	Ribosomal small subunit biogenesis	3	70	1.88	0.0014
	Molecular Function (Gene Ontology)					
1	GO:0003735	Structural constituent of ribosome	9	159	2.0	3.57×10^{-14}
2	GO:0003723	RNA binding	9	1649	0.99	1.11×10^{-5}
3	GO:0019843	rRNA binding	3	62	1.93	0.0046
4	GO:1990948	Ubiquitin ligase inhibitor activity	2	6	2.77	0.0052
5	GO:1901363	Heterocyclic compound binding	10	5831	0.48	0.0180
6	GO:0097159	Organic cyclic compound binding	10	5916	0.48	0.0184
STRING database data are presented						

Table S11: Primers and probes were designed for this study.

rs4655707<br>*SERBP1*	5′- TCAATCACTTCACATAACCTCCA-3′<br>5′- ATCATTTGCAAGATAATTGTCAGGT-3′

	5′-FAM- CAGGGCACAGC**T**TAGGCCA-RTQ1-3′<br>5′-ROX- CAGGGCACAGC**C**TAGGCCA-BHQ2-3′
rs1058074<br>*SERBP1*	5′- AGCCAGTTTGTCAATCAAAAG-3′<br>5′- CTAATGACTTAATTGGGAAAGTTGAA-3′<br>5′- FAM-AGTGACAAT**T**AGATTAAATCC-RTQ1-3′<br>5′- ROX-AGTGACAAT**C**AGATTAAATCC-BHQ2-3′
rs12561767<br>*SERBP1*	5′- GCATGCCTGTAGTCCCAGTA -3′<br>5′- GTCTCCTGTCTCCCATGCTA-3′<br>5′- FAM-CTTGAACTTCA**G**AATGGCCAAAATC-RTQ1-3′<br>5′- ROX-CTTGAACTTCA**A**AATGGCCAAAATC-BHQ2-3′
rs12566098<br>*SERBP1*	5′- CAGTTGTATGACAATGGTAAAATGA -3′<br>5′- CTCCCAAAGTGCTGGGATTA -3′<br>5′-FAM- CACATAAA**C**CACTCTTTTAAG- RTQ1-3′<br>5′-ROX- CACATAAA**G**CACTCTTTTAAG-BHQ2-3′
rs6702742<br>*SERBP1*	5′-CCATCCCATAAGACATTATCTTGTAA-3′<br>5′- GGGGAACTGTCAAAGACGAA-3′<br>5′- FAM-TATGGATTA**A**CCTCTAGTTA-RTQ1-3′<br>5′- ROX-TATGGATTA**G**CCTCTAGTTA-BHQ2-3′
Primers and probes were designed in the Laboratory of Genomic Research (Research Institute for Genetic and Molecular Epidemiology, Kursk State Medical University).	
